# Quantification of aortic pulse wave velocity from a population based cohort: a fully automatic method

**DOI:** 10.1186/s12968-019-0530-y

**Published:** 2019-05-13

**Authors:** Rahil Shahzad, Arun Shankar, Raquel Amier, Robin Nijveldt, Jos J. M. Westenberg, Albert de Roos, Boudewijn P. F. Lelieveldt, Rob J. van der Geest, on behalf of the Heart Brain Connection study group

**Affiliations:** 1 Department of Radiology, Leiden University Medical Center, Albinusdreef 2, Leiden, 2333 ZA The Netherlands; 2 Department of Cardiology, VU University Medical Center, De Boelelaan 1117, Amsterdam, 1081 HV The Netherlands; 3 Intelligent Systems Department, Delft University of Technology, Van Mourik Broekmanweg 6, Delft, 2628 XE The Netherlands

**Keywords:** Pulse wave velocity, Velocity encoded MRI, Image registration, Centerline estimation, Multi-atlas-based segmentation

## Abstract

**Background:**

Aortic pulse wave velocity (PWV) is an indicator of aortic stiffness and is used as a predictor of adverse cardiovascular events. PWV can be non-invasively assessed using magnetic resonance imaging (MRI). PWV computation requires two components, the length of the aortic arch and the time taken for the systolic pressure wave to travel through the aortic arch. The aortic length is calculated using a multi-slice 3D scan and the transit time is computed using a 2D velocity encoded MRI (VE) scan. In this study we present and evaluate an automatic method to quantify the aortic pulse wave velocity using a large population-based cohort.

**Methods:**

For this study 212 subjects were retrospectively selected from a large multi-center heart-brain connection cohort. For each subject a multi-slice 3D scan of the aorta was acquired in an oblique-sagittal plane and a 2D VE scan acquired in a transverse plane cutting through the proximal ascending and descending aorta. PWV was calculated in three stages: (*i*) a multi-atlas-based segmentation method was developed to segment the aortic arch from the multi-slice 3D scan and subsequently estimate the length of the proximal aorta, (*ii*) an algorithm that delineates the proximal ascending and descending aorta from the time-resolved 2D VE scan and subsequently obtains the velocity-time flow curves was also developed, and (*iii*) automatic methods that can compute the transit time from the velocity-time flow curves were implemented and investigated. Finally the PWV was obtained by combining the aortic length and the transit time.

**Results:**

Quantitative evaluation with respect to the length of the aortic arch as well as the computed PWV were performend by comparing the results of the novel automatic method to those obtained manually. The mean absolute difference in aortic length obtained automatically as compared to those obtained manually was 3.3 ± 2.8 mm (*p < 0.05*), the manual inter-observer variability on a subset of 45 scans was 3.4 ± 3.4 mm (*p = 0.49*). Bland-Altman analysis between the automataic method and the manual methods showed a bias of 0.0 (-5.0,5.0) m/s for the foot-to-foot approach, -0.1 (-1.2, 1.1) and -0.2 (-2.6, 2.1) m/s for the half-max and the cross-correlation methods, respectively.

**Conclusion:**

We proposed and evaluated a fully automatic method to calculate the PWV on a large set of multi-center MRI scans. It was observed that the overall results obtained had very good agreement with manual analysis. Our proposed automatic method would be very beneficial for large population based studies, where manual analysis requires a lot of manpower.

## Background

Pulse wave velocity (PWV) of the aorta is an indication of aortic stiffness [1, 2]. PWV is defined as the propagation speed of the pressure wave along a vessel segment [3]. Aortic stiffness increases with age and can also be caused by a number of cardiovascular diseases [4]. A number of population based studies have been conducted to study the relationship between aortic stiffness as measured by PWV and age [5–7], ethnicity [8, 9] and, future cardiovascular events [10, 11]. Recent studies have shown that PWV changes with age are more prominent in subjects with Marfan syndrome than in healthy volunteers [12]. PWV measurement using Magnetic resonance imaging (MRI) is widely used as a non-invasive technique and has been well validated [3, 5, 13, 14]. The most common method to quantify aortic PWV is by calculating the length of the aorta and the transit time i.e. the time taken for the systolic wave to propagate from one reference point within the aorta to another [15]. The length of the aorta is either derived from a single-slice (2D), or a multi-slice (3D) oblique-sagittal scan parallel to the aortic arch. In clinical practice the length is generally obtained manually, either on the single-slice 2D oblique-sagittal scan or a maximum intensity projected (MIP) image from the multi-slice 3D scan. The 2D approximation is fast and less labour intensive. However, it does not provide an accurate representation of the complex aorta, which can at times be tortuous. The transit time is calculated from a through-plane velocity encoded (VE) MRI scan with high temporal resolution acquired along the cross-section of the aorta. Computing the transit times requires segmenting the ascending and descending aorta in the VE scans [16–18]. These methods generally require manual interaction. Literature provides a number of techniques to estimate the arrival times of blood flow at the two locations of the aorta using the flow curves [15, 19–21]. To the best of our knowledge, literature presents three main categories of studies related to PWV analysis, (*i*) extraction of the aorta length from the scout scan, (*ii*) segmentation of the aorta using the 2D+t through-plane VE scan, and (*iii*) the flow curve analysis. Studies that present new methods of computing PWV are predominantly semi-automatic and rely significantly on manual interactions. Thus, methods that reduce the subjectivity in measurements and improve the overall time and efficiency of calculation are limited. The aim of this study is to develop and validate a fully-automatic technique for computation of the aortic arch PWV. Having such a method would be beneficial for conducting automated analysis on large population based studies [22–24]. Our proposed method has three main stages: (*i*) segmenting the aortic arch in 3D for calculating the length of the aorta, (*ii*) detection and propagation of the 2D aorta contours for computing the time-velocity flow curves, and (*iii*) estimating the transit time for calculating the PWV.

## Methods

### Study population

For this work, a total of 212 subjects were randomly identified from an existing database of prospectively included patients with carotid occlusive disease, heart failure, vascular related cognitive impairment, and healthy controls (134 men, mean age 68.5 ± 8.4 years). The data used in our study is part of a large multi-center heart-brain connection cohort [22]. Four medical centers situated in The Netherlands: VU Medical Center (VUMC), Leiden University Medical Center (LUMC), Maastricht University Medical Center (MUMC) and University Medical Center Utrecht (UMCU) were involved. Various scans for both the brain and the heart were acquired at each of the centers. For details about the scans acquired and the selection criteria for the subjects the readers are referred to the publication of van Buchem et al. [22] and to the study design publication of Hooghiemstra et al. [25]. The institutional review board approved this study, and all patients were informed and provided written consent. For the current study two MRI scan types from the cohort were used. Each of the subjects had a multi-slice 3D scan of the aorta acquired in an oblique-sagittal plane and a VE scan acquired transversally to the proximal aorta. Table 1 presents the characteristics of the subjects. Note that, the subjects are categorized as belonging to four different groups, subjects with carotid occlusive disease, heart failure, vascular related cognitive impairment, and healthy controls.

**Table 1:** Population characteristics

Variable	Value
Sample size	212
*Men*	134 (63%)
Age, range	68.5 (51–91)
Controls	37 (17%)
COD	41 (19%)
HF	67 (32%)
VCI	67 (32%)

The included subjects fall in four categories: healthy controls, subjects with carotid occlusive disease (COD), subjects with vascular related cognitive impairment (VCI) and subjects with heart failure (HF)

### MRI acquisition

**Fig. 1:**
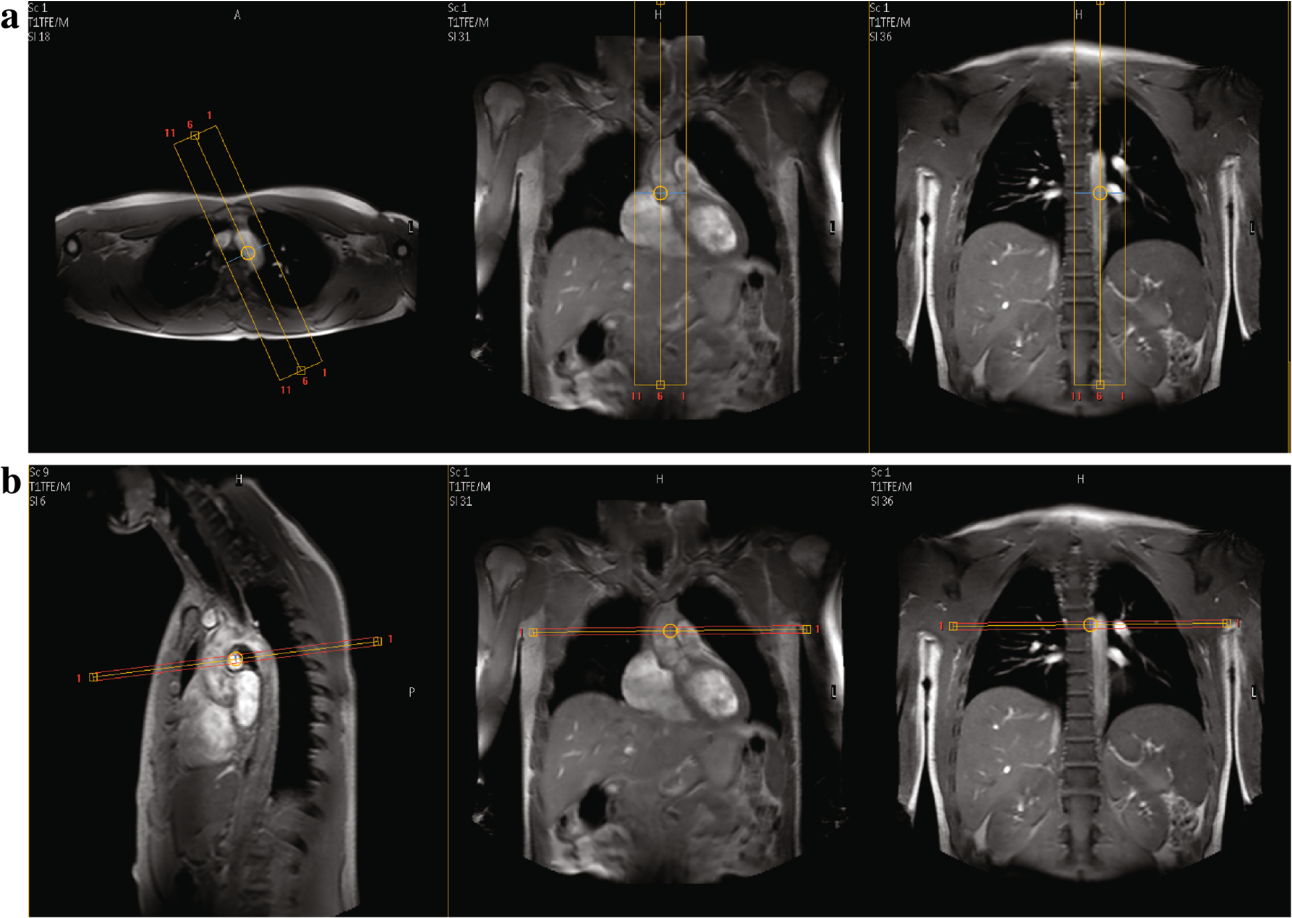
Planning of the multi-slice 3D scan (**a**) and the VE scan (**b**)

Each center used a 3T MRI scanner from Philips Medical System (Best, The Netherlands). However, the scanner models were different: LUMC used an Ingenia 3.0T, MUMC, UMCU and VUMC used an Achieva 3.0T. Each center was instructed to follow the same protocol to scan the subjects. The multi-slice 3D scans was acquired using a T1 gradient echo-imaging mode without any ECG or respiratory gating. The reconstructed pixel size was 1.76 × 1.76 mm^2^ and a slice thickness of 5 mm. The flip angle was 15 ^∘^, TR/TE = 4.8/2.4 ms. The reconstructed image matrix size was 256 × 256 × 15. Average acquisition time for the multi-slice 3D scan was 9 s. The VE scan was acquired with a through-plane velocity encoding, with a phase contrast velocity encoding value (VENC) between 1.5–2.0 m/s. These scans were acquired using the sensitivity encoding protocol (SENSE) [26], with a SENSE acceleration factor of 1.5. The scans were obtained with free breathing and were retrospectively ECG triggered. The acquired VE scan is non-segmented and has an average temporal resolution of 9.8 ms. The field-of-view was 320 mm, scan matrix was 128 × 128, slice thickness was 8 mm, flip angle was 10 ^∘^, TR/TE = 4.7/2.8 ms. Images were reconstructed to a pixel size of 1.25 × 1.25 mm and a reconstructed phase interval of 5 ms, resulting in average between 140–250 cardiac phases, depending on the heart rate. Average acquisition time for the VE scan was 2 min. Figure 1 shows the planning of the multi-slice 3D scan and the subsequent VE scan.

### Scan quality check

To ensure that the acquired MRI scans were of sufficient diagnostic quality and did not deviate from the defined protocol. An observer (RA) visually inspected all the scans from each of the centers to check for any discrepancy (incorrect planning, extreme breathing or interference artefacts, aliasing, etc.). After the quality check 11 subjects were found to have insufficient diagnostics quality scans had were excluded from further analysis.

### Proposed automatic method

The proposed fully automatic method has three main stages: (***i***) segmenting the aorta in 3D using the multi-slice 3D scan for calculating the length of the aorta, (***ii***) detection and propagation of the 2D aorta contours on the multi-phase VE scan for computing the time-velocity flow curves, and (***iii***) estimating the transit time from the obtained time-velocity flow curves. The PWV is computed by combining the length of the aorta and the transit time. For our automatic method no intermediate user interaction is required between the three main stages in the workflow. For preparation of the data, the scan sessions need to be organized into relevant folders, our method automatically picks out the relevant scans based on the naming conventions for computing the aorta length and computing the flow curves. The final output of our method are quantitative numbers such as, aorta length, arrival times of the pressure wave at the ascending and descending aorta, transit time and the pulse wave velocity. The above-mentioned three stages consist of a number of intermediary steps that are explained in the following two sections.

### Computation of the aortic arch length

**Fig. 2:**
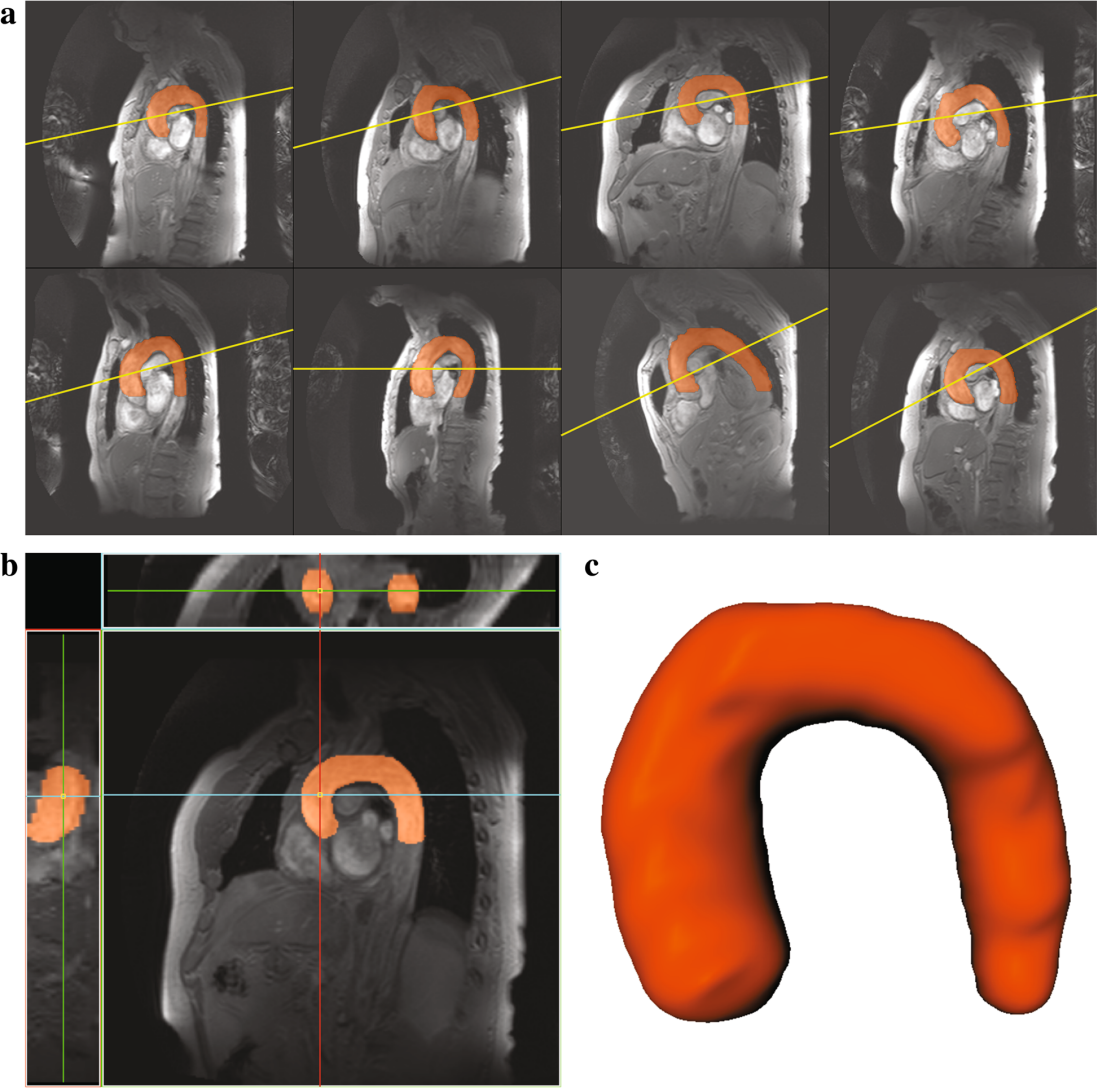
The 8 atlas multi-slice 3D scans with the aorta labels overlaied in orange (**a**), the yellow line indicates the cutting plane of velocity encoded MR scan. One of the atlas scans with its sagittal, coronal and axial views (**b**). The atlas label visualized as a 3D surface (**c**)

The length of the aorta was computed by first segmenting the aortic arch and then obtaining the centerline from it. A multi-atlas-based segmentation [27] approach was developed to segment the aorta in 3D from the multi-slice 3D scans. Eight additional subjects from the multi-center hear-brain connection cohort that are not part of the randomly selected 212 subjects were used as atlas scans and were selected based on the following properties: (*i*) the inherent shape of the aorta, (*ii*) the orientation and position of the aorta within the scan’s field of view (FOV), and (*iii*) the image quality of the scan. These atlases were carefully selected such that they are representative of the entire population. For each atlas scan, the proximal aorta was carefully delineated by an experienced user (AS, with supervision from RS). Figure 2 shows the atlas scans and the annotated 3D aorta label. It should be noted that the aorta segmentation covers a larger region i.e. it extends below the VE scanning plane. This was done to compensate for the variation of the VE scan planning (yellow line in Fig. 2). Multi-atlas-based segmentation consists of three steps. First, each of the multi-slice 3D atlas scans were registered [28, 29] to the unseen subjects’ multi-slice 3D scans. In the registration procedure, the transformation parameters $\hat {\mathbf {T}}$ that minimize the dissimilarity $\mathcal {C}(\mathbf {T}; F, M)$ between the fixed image (*F*) and the moving image (*M*) are determined. The optimization problem can be mathematically represented as:

1 $$ \hat{\mathbf{T}} = \text{arg} \operatorname*{min}_{\mathbf{T}} \mathcal{C} \left(\mathbf{T}; F,M\right).   $$

Second, the resulting transformation $\hat {\mathbf {T}}$ from each of the atlas registration was used to map the aorta label from atlas scans (*F*) onto the subjects’ scan (*M*). In our case, we have eight unique aorta labels mapped for each subject. Third, the final aorta segmentation was obtained by combining the transformed labels by using a label fusion strategy [30]. A number of experiments were performed for atlas selection, registration optimization and label fusion strategy. These experiments are described in “Multi-atlas based segmentation” section. From the obtained aorta segmentation the centerline of the aorta $\mathcal {V}=\left \{\mathbf {v}_{i=\{1,\dots,n\}}\right \}$, represented by a set of spatial points **v**_*i*_, were extracted. This was done by skeletonizing the segmentation by applying a 3D homotopic thinning algorithm [31]. A subsequent pruning step was performed to remove any unwanted side branches that may arise due to an uneven segmented surface. The longest connected graph was retained as the centerline. Finally, the length of the aorta defined from the proximal ascending to the descending aorta was calculated. This was done by estimating the cutting plane of the VE scan through the multi-slice 3D scan. The equation of a plane is defined as:

$$\begin{array}{*{20}l} &ax+by+cz=d\\ &\mathrm{or:}\\ &\overrightarrow{m}.\overrightarrow{n} = d,  \end{array} $$

where $\overrightarrow {m}=(x,y,z)$ and $\overrightarrow {n}=(a,b,c)$. A centerline point $\mathbf {v}_{i}\in {\mathcal {V}}$ is retained if,

$$\left\lbrace i : (\overrightarrow{\mathbf{v}_{i}}.\overrightarrow{n})\geq d\right\rbrace. $$

**Fig. 3:**
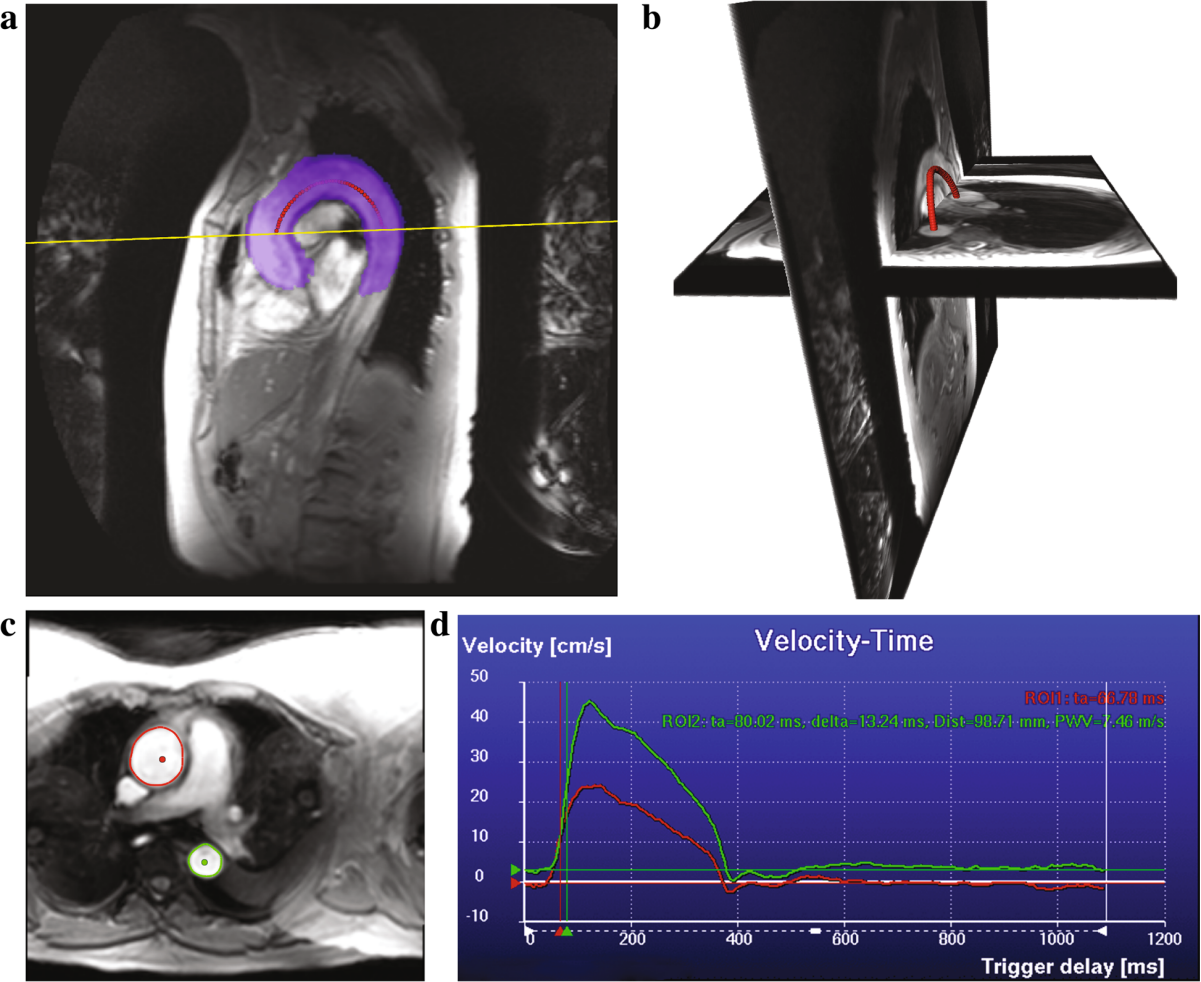
The resulting segmentation of the aorta for one of the subjects after multi-atlas-based segmentation (in blue) and the calculated centerline (in red) on the multi-slice scan (**a**). The multi-slice scan and the VE scan with the extracted centerline represented in 3D (**b**). Automatically delineated ascending and descending aorta contours on the magnitude VE scan, the two dots indicate the centerline point that were used as initiation (**c**). The computed flow curves obtained from the phase VE scan (**d**), red curve is the velocity-time curve for the ascending aorta (ROI1) and the green curve is the velocity-time curve for the descending aorta (ROI2), ta stands for arrival time, delta stands for transit time, Dist is the length of the aortic arch and PWV is the pulse wave velocity

The length $\mathcal {L}$ was then estimated by summing the vector lengths between all the retained points using Dijkstra’s shortest path [32]. Figure 3 a-b shows an example of the calculated centerline for one of the subjects.

### Multi-atlas based segmentation

A number of preliminary experiments were conducted to obtain the registration parameters for the multi-atlas-based segmentation. These experiments were performed on the atlas scans using a leave-one-out strategy. From these experiments it was observed that a three-stage registration approach with a multi-resolution coarse-to-fine strategy with three resolutions resulted in the best performance. First, an affine registration was performed to roughly align the atlas scan (fixed image) and the subjects’ scan (moving image) globally. Second, using the transformation results of the first step as an initiation, another affine registration was performed. In this stage a fixed aorta mask was used. The aorta mask was generated by dilating the aorta label on the atlas scan by two voxels in-plane. This registration step helps to locally align the aorta between the fixed and the moving image. Finally, a non-rigid B-spline registration was performed using the transformation from the previous stage as initialization. A fixed aorta mask was also used in this stage. For all three stages mutual information was used as the similarity measure and adaptive stochastic gradient descent [33] was used for optimization. The number of iterations was set to 512 for the affine transformations and 2048 for the B-spline transformation. A B-spline grid spacing of 15 mm was used with a pyramid schedule. Registrations were performed using the publicly available registration software elastix [34]. For automatic contour propagation the temporal VE image that was used to detect the initial contour was chosen to be timepoint 30, which corresponds approximately to the moment of peak flow velocity. Initial experiments and our previous experience indicated that this time point has sufficient image contrast for accurate aorta contour detection.

### Computing the flow curves and transit time

The time-velocity flow curves were obtained based on the method proposed by van der Geest et al. [35] The method has three main steps: (*i*) initial contour detection, (*ii*) contour propagation, and (*iii*) deriving the flow parameters. The initial contours for the ascending and descending aorta are detected on one of the 2D+t VE scans, this is ideally computed on a temporal image which has good image contrast. The vessel boundary detection is initialized from the centerline point on the ascending and descending aorta. These centerline points were automatically obtained from the previous step. Where, along with the calculated aorta length, the two centerline points from the proximal ascending and descending aorta that lie on the VE plane were also estimated. Starting from the centerline point evenly spaced angular radial scan lines with a fixed length are constructed to detect pixels that have the highest edge response. A minimum cost contour detection algorithm was used to delineate the contours through the edge pixels, which ensures a smooth contour through the detected vessel edge boundary. Propagating the detected contours through the remaining cardiac phases followed this step. This was done by using a previously validated multi-dimensional dynamic programming approach which ensures temporal continuity of the aortic contours [35, 36]. The time-velocity flow curves for the ascending and descending aorta were computed by calculating the mean velocity within the detected contours throughout the cardiac phases. From these curves the arrival times at the ascending and descending aorta can be automatically determined to compute the transit time *Δt*. A number of different methods are described in literature which can compute the transit times from the time-velocity flow curves [15]. Our proposed method can compute the transit times using three popular techniques, foot-to-foot method [37], the half-max method [38], and cross-correlation method [39]. Finally the PWV is computed as:

$$\text{PWV}=\mathcal{L}/\Delta t. $$

Figure 3 c-d shows an example of the contour detection step and the computed flow curves for one of the subjects.

### Manual analysis

**Fig. 4:**
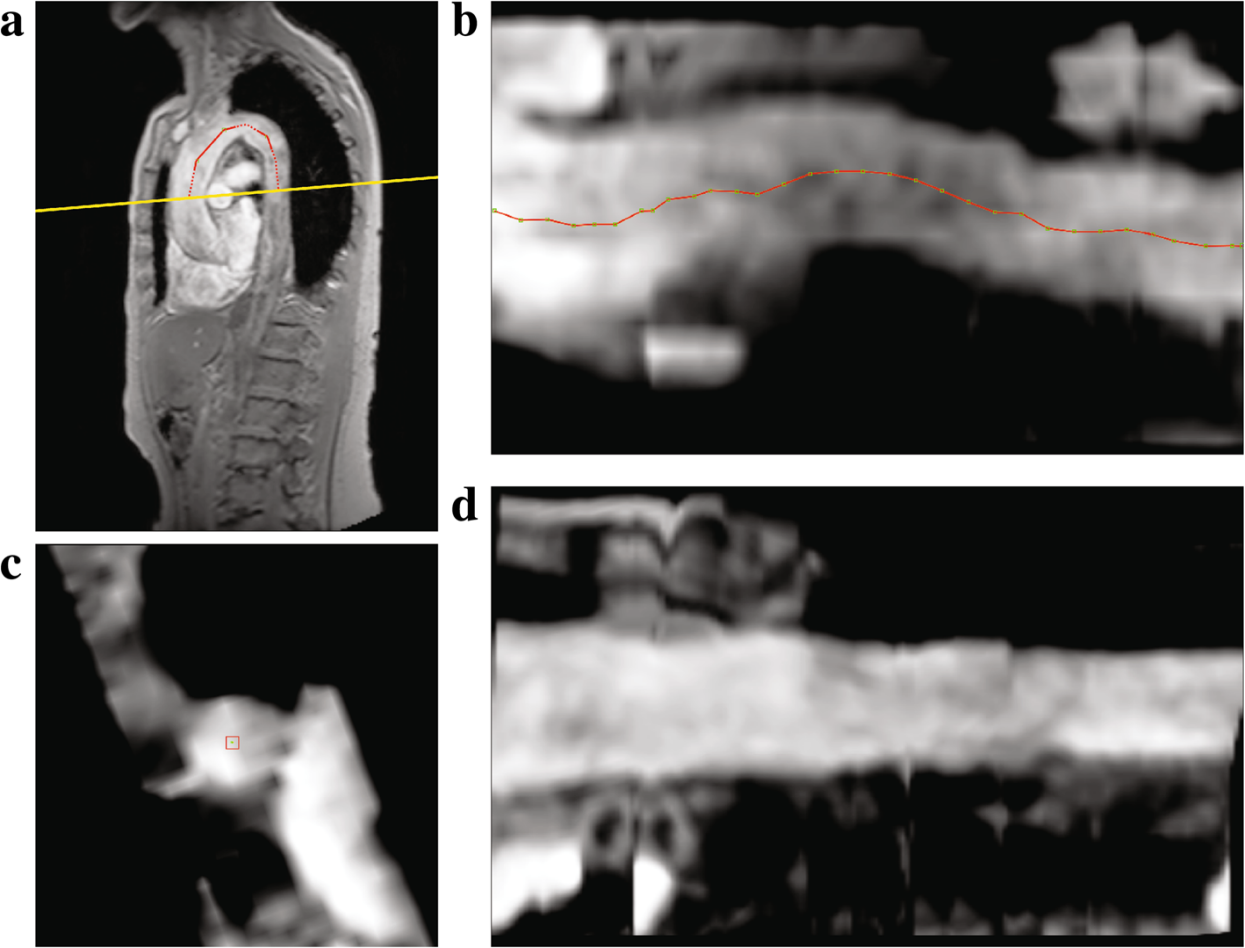
The process of obtaining the manual centerline length. Initial centerline is obtained by tracing a poly-line on the multi-slice scan (red), dotted line indicates that the anchor points are out-of-plane (**a**). Multi-planer reformatted (MPR) image obtained using the initial centerline, sampled at a distance of 1 mm (overlaid in red) (**b**). Refining the centerline on the MPR axial slice by dragging points (red) in the middle of the lumen when required (**c**). MPR image stack generated using the refined centerline points (**d**)

**Fig. 5:**
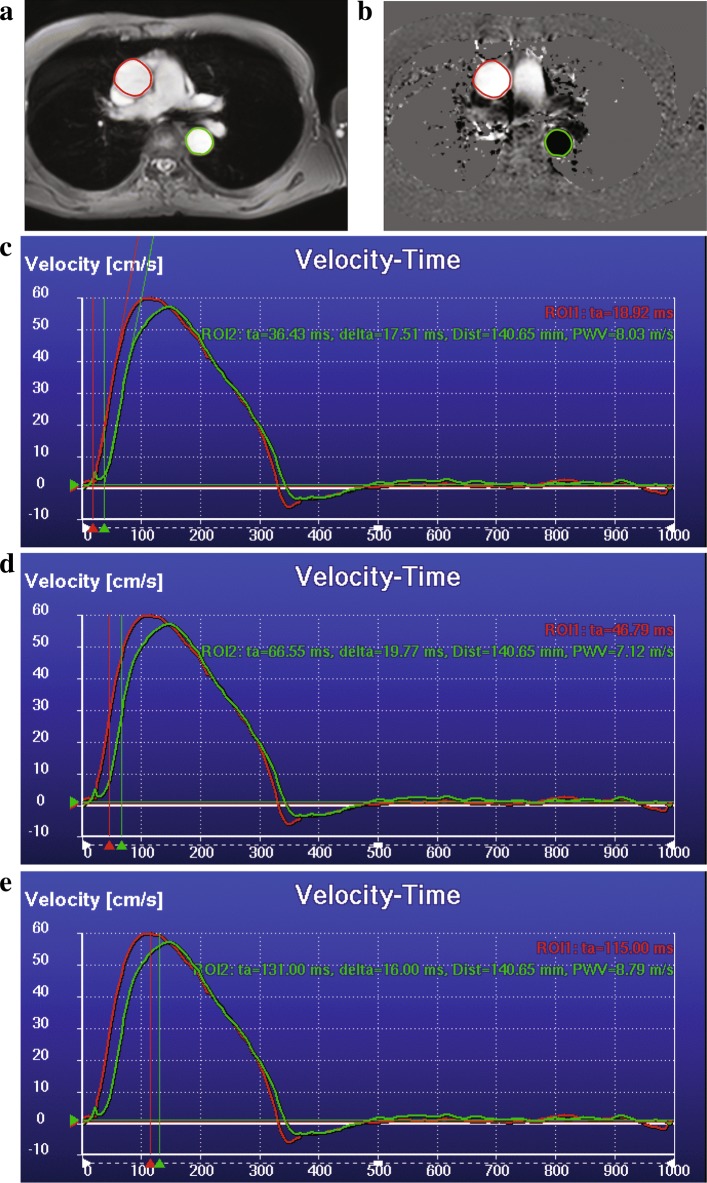
Process of obtaining the manual flow curves. Delineated magnitude VE scan (**a**). Velocity VE scan with the manual contours (**b**). Velocity-Time curve computed using the contours and the velocity VE scan, transit time computed using the foot-to-foot method(**c**) using the half-max method (**d**) and the cross-correlation method (**e**). For the time velocity curves, x-axis is the trigger delay in ms and y-axis is the velocity in cm/s. Red curve is the velocity-time curve for the ascending aorta (ROI1), the green curve is the velocity-time curve for the descending aorta (ROI2), ta stands for arrival time, delta stands for transit time, Dist is the length of the aortic arch and PWV is the pulse wave velocity

To compare the performance of the proposed automatic method, the results of the automatic PWV quantifications were compared to those obtained manually. The values were obtained using the software MASS (LUMC, The Netherlands). The manual computations were performed in two stages. Firstly, the centerline of the proximal aorta was manually assessed. This was done by initially drawing a rough centerline through the multi-slice 3D scan. Using the initial centerline, a multi-planar reformatted (MPR) image stack with a slice thickness of 1 mm was computed. The centerline was then refined on the cross-sectional MPR stack by clicking points in the center of the vessel lumen. The refined centerline was used to compute the length of the aorta. This process is presented in Fig. 4. It can be appreciated that the MPR stack obtained using the refined centerline Fig. 4d is much more parallel than the initial one Fig. 4b, which signifies that the refined centerline is indeed in the center of the vessel lumen. Thus, the obtained 3D centerline length is very accurate. Secondly, the contours for the proximal ascending and descending aorta on the VE scan were semi-automatically obtained. This was done by manually initializing two contours for the ascending and descending aorta on one of the cardiac phases of the magnitude images of the VE scan, followed by automated propagation over the remaining phases. Subsequently, the contour segmentation results were reviewed and the user had the ability to modify the contours to fit the aorta over multiple phases. These contours were then used to compute the flow velocity-time curves and derive the transit times using the three curve analysis methods i.e. the foot-to-foot, half-max and the cross-correlation method. Figure 5 shows one such example.

## Results

A visual check indicated that out of 201 subjects the centerline detection step failed on 3 subjects. Additionally, the contour propagation step failed on 6 subjects.

### Aortic length

**Fig. 6:**
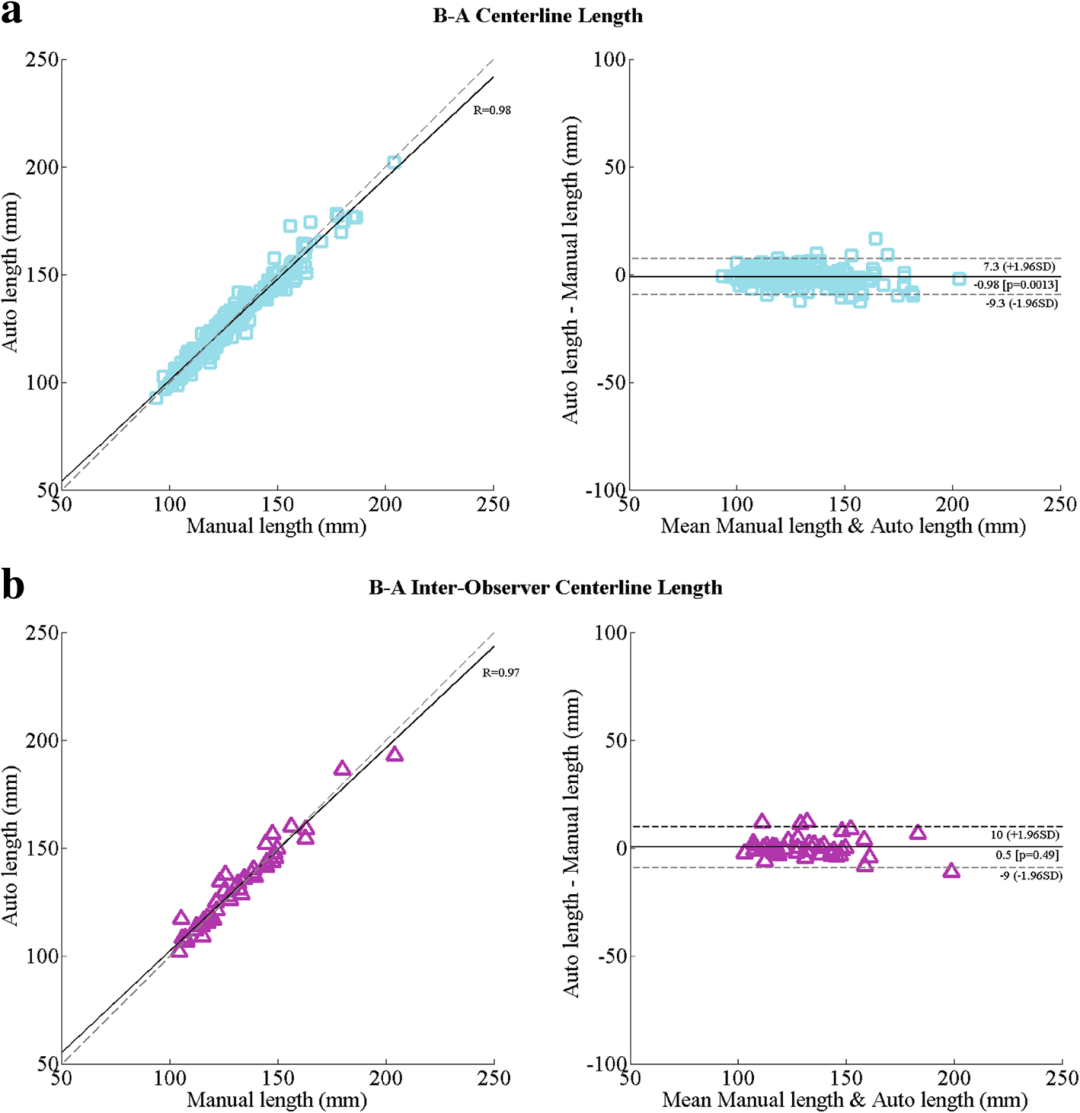
Correlation and Bland-Altman plots for computing the centerline length, between the automatic method and the manual approach (**a**), the inter-observer comparison (**b**). For the correlation plots (left) solid line indicates the line of best fit and the dashed line shows the diagonal reference line. For the Bland-Altman plot (right) the solid line shows the bias and the dashed lines show the 95% confidence interval

The automatically detected centerline length was compared to those obtained manually on a set of 198 subjects. Bland-Altman analysis indicated that the bias was -1.0 (-9.3, 7.3) mm. It was also observed that the mean absolute difference in the length was 3.3 ± 2.6 mm. An inter-observer analysis for manual computation of the aorta lenght (as described in “Manual analysis” section) was also conducted on a set of 45 randomly selected subjects. It was observed that the mean absolute aortic length difference between the observers was 3.4 ± 3.4 mm with a bias of 0.5 (-9.0, 10.0) mm. More detailed results are presented in Table 2 and the correlation plots are provided in Fig. 6.

**Table 2:** Performance of the proposed automatic method

Measure	N	Manual	Automatic	*R* (CI for *β*)	B-A (95% CI)	Abs diff
Centerline length (mm)	198	131.6 ± 20.2	130.6 ± 19.5	0.98 (0.97, 0.98)	-1.0 (-9.3, 7.3)	3.3 ± 2.8
*Inter-observer centerline length* (mm)	45	132.0 ± 20.8^*#*^	132.5 ± 20.1^&^	0.97 (0.95, 0.99)	0.5 (-9.0, 10)	3.4 ± 3.4
Transit time *foot-to-foot* (ms)	186	16.7 ± 8.2	16.0 ± 5.7	0.98 (0.97, 0.98)	-0.1 (-2.4, 2.1)	0.9 ± 2.4
Transit time *half-max* (ms)	192	16.4 ± 5.0	16.5 ± 5.0	0.99 (0.99, 0.99)	0.1 (-1.1, 1.2)	0.4 ± 0.5
Transit time *cross-correlation* (ms)	192	14.4 ± 4.6	14.5 ± 4.7	0.97 (0.97, 0.98)	0.1 (-1.9, 2.2)	0.5 ± 0.9
Pulse wave velocity *foot-to-foot* (m/s)	186	9.5 ± 5.7	9.5 ± 5.2	0.89 (0.86, 0.92)	0.0 (-5.0, 5.0)	0.8 ± 0.9
Pulse wave velocity *half-max* (m/s)	192	8.8 ± 3.3	8.7 ± 3.4	0.99 (0.98, 0.99)	-0.1 (-1.2, 1.1)	0.4 ± 0.5
Pulse wave velocity *cross-correlation* (m/s)	192	10.4 ± 5.8	10.2 ± 5.5	0.98 (0.97, 0.98)	-0.2 (-2.6, 2.1)	0.7 ± 1.0

N is the number of measurments, Manual is the measurements obtained using the manual method (mean ±SD), Automatic is the measurement obtained using the proposed automatic method (mean ±SD), *R* is the Pearson correlation coefficient along with the linear regression *β* confidence interval (CI). B-A is the Bland-Altman bias along with the 95% CI. Abs diff is the average absolute difference between the manual and automatic measurements (mean ±SD). ^*#*^ Observer 1 and ^&^ Observer 2

### Transit-time analysis

The transit-time between the automatic method and the manual method were compared on 192 subjects. The transit times for all three methods i.e. the foot-to-foot, cross-correlation and the half-max methods have been compared. It was noticed that the foot-to-foot method failed to accurately detect the foot of the curve for an additional 6 subjects. Bland-Altman analysis between the proposed automatic method and the manual analysis for the transit time (*Δt*) showed that the foot-to-foot method had a bias of -0.1 (-2.4, 2.1) ms, while the half-max method had a bias of 0.1 (-1.1, 1.2) ms and the cross-correlation method had a bias of 0.1 (-1.9, 2.2) ms. See Table 2 for more detailed analysis.

### PWV computation

**Fig. 7:**
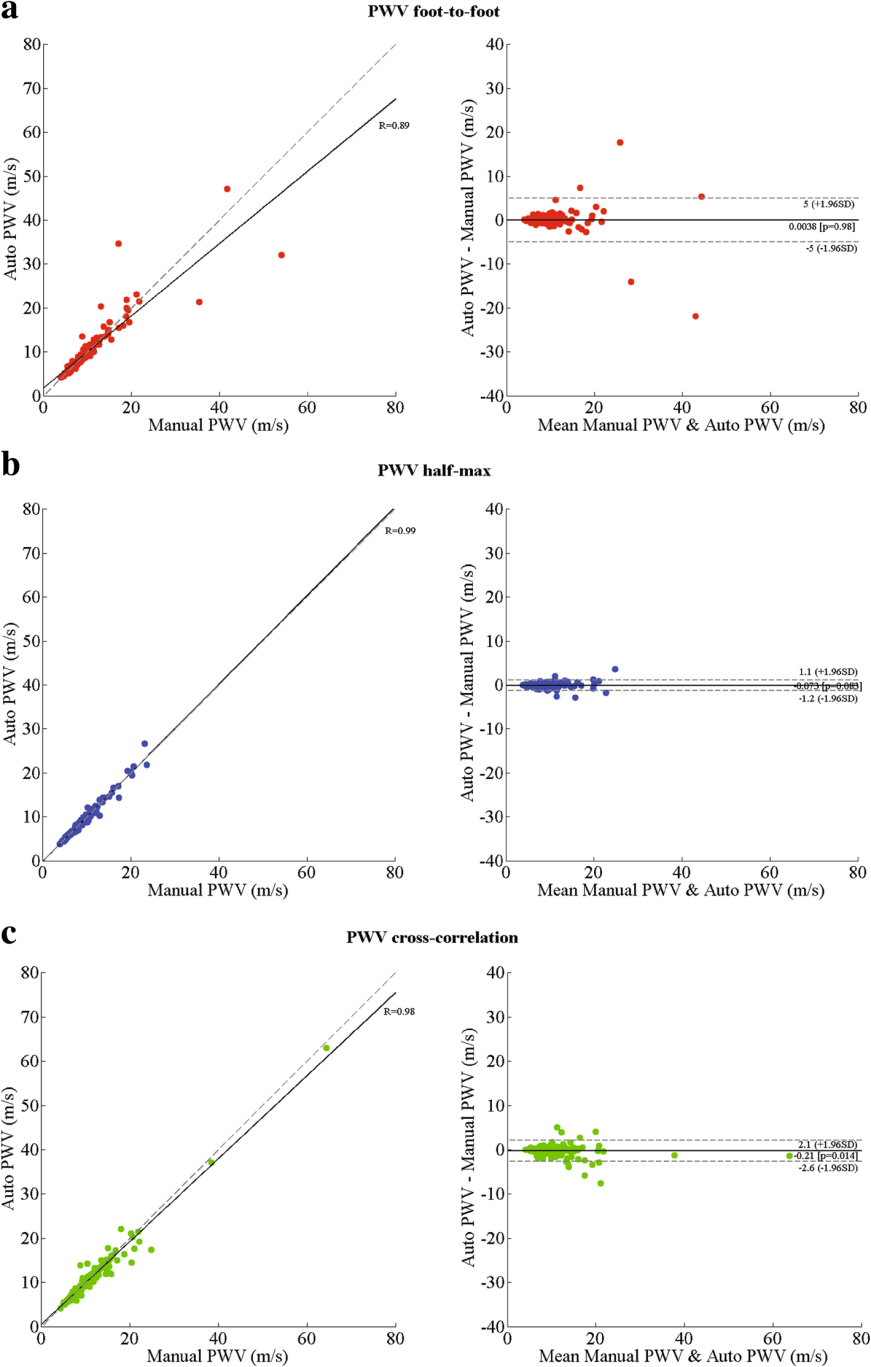
Correlation and Bland-Altman plots for the PWV computation between the automatic method and manual computations, foot-to-foot method (**a**), half-max method (**b**), and cross-correlation (**c**).For the correlation plots (left) solid line indicates the line of best fit and the dashed line shows the diagonal reference line. For the Bland-Altman plot (right) the solid line shows the bias and the dashed lines show the 95% confidence interval

Bland-Altman analysis between the automatic method and the manual method for the PWV showed a bias of 0.0 (-5.0, 5.0) m/s (on 186 subjects), bias of -0.1 (-1.2, 1.1) m/s and bias of -0.2 (-2.6, 2.1) m/s for the foot-to-foot, half-max, and the cross-correlation methods, respectively. The mean absolute difference between the automatic computations and the manual one for the PWV was 0.8 ± 0.9, 0.4 ± 0.5 and 0.7 ± 1.0 m/s for the three methods, respectively. Detailed results including correlation and Bland-Altman analysis for both, the transit times and PWV are presented in Table 2. Additional plots are presented in Fig. 7.

## Discussion

We have presented a fully automatic method that can compute the length of the aorta in 3D on a multi-slice 3D scan, segment the ascending and descending aorta from the 2D velocity-encoded MRI (VE) scans, compute the time-velocity curves, and estimate the pulse wave velocity (PWV). When compared to the manual technique, our proposed method shows excellent agreement.

The average error of the computed aorta length which was 3.3 ± 2.8 mm, which is smaller than the slice thickness of the acquired multi-slice scan which was 5 mm. A 2D aorta length calculation approach would result in an error, which can be far greater than the one our automatic method generates. It can also be observed from the inter-observer analysis that the 3D aorta length error is within the inter-observer variability (3.4 ± 3.4 mm). The transit time computation for the three different approaches show good agreement, which implies that the initialized contours on the VE scan and the computed flow curves are very accurate. Transit times and PWV comparison showed very small absolute differences that indicate that our proposed method is quite robust.

### Previous work

The method presented by van Engelen et al. [42] also extracts the aorta length in 3D, by using a scan protocol for volumetric acquisition. Their method is based on a vesselness filter [40] and a minimum-cost path [41] approach to extract the centerline. The response of a vesselness filter is generally good only on images with high scan quality and sufficient contrast difference between the aorta lumen and the surrounding tissues (angiographic or black blood images). The multi-slice 3D scan protocol used in our study was optimized to shorten the duration of the scan time which often leads to poor signal-to-noise ratio and also the image resolution is inferior when compared to the volumetric protocol used in the study of van Engelen et al. Hence using a vesselness filter to preprocess our data would not give a good filter response. Moreover, comparing the results obtained by van Engelen et al. [42] on the 3D volumetric scan protocol shows that they achieved an absolute centerline length error of 4.8 mm, whereas our fully automatic method results in a much smaller error of 3.3 mm. The method of Babin et al. [43] uses graph paths with intensity related information and Dijkstra’s shortest path [32] to extract the centerline. Both methods presented above require manual start and end points to compute the centerline length. Whereas our proposed method not only computes the 3D aorta length automatically, but also calculates the flow characteristics without any need for user interaction.

### Different approaches for computing transit time

For automatic computation of the transit time (*Δt*) we implemented the three popular velocity-time curve analysis techniques described in literature, foot-to-foot method [37], the half-max method [38], and the cross-correlation method [39]. Previous studies have compared these three methods to investigate which of them is the most accurate method that can estimate PWV non-invasively from MRI scans as compared to the invasive pressure wire measurement [19, 21]. A complete and thorough analysis of which of these three methods is superior is out of scope of this manuscript. A previous study [13] which compares the PWV computation using the invasive pressure wire measurement to the non-invasive MRI based measurement using the foot-to-foot manual approach for analyzing the flow curves showed excellent agreement between the two approaches. However, as per the observations of our automatic analysis we noticed that the foot-to-foot method completely failed on 6 subjects and also had a lower Pearson correlation coefficient, due to the inability of the method to reliably detect the feet of the curves accurately. The relatively lower temporal resolution of the VE scan as compared to pressure wire measurements [44] makes it rather difficult to accurately detect the foot of the velocity-time curves automatically.

Also, the VE scans suffer from lower signal intensities and artefacts in the initial few time points of the scan. This results in noisy velocity-time curves that add to the difficulty of automatically detecting the foot. The cross-correlation method on the other hand does not look at the feet of the velocity-time curves but takes the entire curve into consideration to estimate the transit time. However, for the computation of *Δt* the arrival-times of the propagating systolic pressure wave are the most important component. By considering the complete curve the cross-correlation method does not differentiate between the up-slope/down-slope components of the curve. Hence this method generally tends to underestimate the *Δt*. The error increases if reflecting waves are present in the descending aorta [39]. The half-max method considers only the up-slope of the curves and computes the arrival-times at the half-max of the detected up-slope component of the curve, which enables the method to only consider the arrival component i.e. the systolic wave and ignore the diastolic part of the curve. Hence this method is more robust in accurately estimating the *Δt* automatically.

From our manual PWV velocity analysis it was also observed that the foot-to-foot method needs manual correction quite often to accurately detect the foot of the curves and the interpretation of the actual foot varies between different observers. Also, the cross-correlation method underestimates the transit-times more often, thus resulting in higher PWV values. The average transit-times from the manual analysis were 16.7, 16.4 and 14.4 ms for the foot-to-foot, half-max and the cross-correlation method, respectively. Figure 4 c-e shows an example case of manually analysed velocity-time curves using the three methods.

### General observations

For a few subject’s we observe PWV values that are higher than 20 m/s. These are unrealistic PWV values even for a cohort of elderly subjects with cardiovascular diseases. Reference PWV values for healthy elderly subjects > 70 years of age is approximately 11.1 ± 4.6 m/s [45]. For subjects suffering with atherosclerosis the PWV values can be higher, 14.9 ± 4 m/s [46]. However, the PWV values can be accurately measured only up to a certain range above which the temporal resolution of the VE scan is not high enough to provide an accurate quantitative measure. From the VE scan protocol used in our study, we estimate this value to be 19 m/s. All subjects with PWV higher than this value should be simply categorized as high PWV. When comparing the three velocity-time curve analysis techniques only on subjects which had a PWV of < 20 m/s, we obtain a Pearson correlation coefficient *R* of 0.95 (on 180 subjects), 0.98 (on 189 subjects) and 0.95 (on 184 subjects) for the foot-to-foot, half-max, and the cross-correlation methods, respectively. The average processing time of our automatic method to analyse each subject is less than 10 min. The computationally expensive part of our method is the aortic arch length calculation, which requires image registration. The time required for registering the 8 atlas scans is approximately 8 min for each subject, the other sub-processes require only a few seconds each. The processing pipeline can also be run in parallel on multiple subjects using computational clusters. This scalability would be really useful for large population based studies.

### Limitations and future work

Our method has a few drawbacks; the atlas-based-segmentation approach requires a representative set of atlases that can cover various aspects of the population. If an acquired scan is very different from the atlases, then it is very likely that the aorta segmentation will fail. Since the registration parameters were not trained to handle such situations the obtained segmentation would be inaccurate. In our analysis this occurred for three subjects, two of them had a field of view (FOV) that was very different to the rest of the scans, and one of them had an anatomically complex shaped aorta. Another drawback of our method is that we assume a reasonably good alignment between the multi-slice scan and the VE scan. As these scans are acquired at different time intervals it could happen that there is patient moment between the two acquisitions and the scans are not well aligned with each other. This causes an initialization error with respect to the centerline points and the location of the aorta. Such a displacement would cause our contour detection step to erroneously segment the wrong vessel-like structure. However, such occurrences are very rare. In the present study this occurred in 6 instances. Moreover, such misalignments would result in an inaccurate PWV even when a manual analysis is performed. Our proposed automatic method had a failure rate of only 3% (i.e. 9 subjects). Our current method lacks the feature to automatically present a confidence measure to estimate the accuracy of the calculated measures, we hope to address this feature in the near future. Currently our method provides a visual representation of the aorta centerline length, the detected contours and the flow curves for each subject. These images can be used to quickly review the results. Apart from calculating the PWV, left ventricular cardiac output can also be calculated using the VE scans [47, 48]. This is done by integrating the flow values within the automatically obtained ascending aorta contour over a complete cardiac cycle and multiplying it with the heart rate. In the present study cardiac output was not computed, as the goal of the study was to evaluate automatic PWV computation.

In the present study we have demonstrated that the 3D length of the aorta can be extracted using the multi-slice 3D scans. Theoretically, this method can be easily extended to other MR protocols used for acquiring scans of the aorta as well. The only requirement would be to select a number of representative scans with accurately segmented aortic arch, to be used as atlases for the multi-atlas-based segmentation approach. Also, our automatic method is not vendor specific. Including atlas scans and sequences from different vendors would enable the method to work on multi-vendor data.

Our method was implemented using freely available software tools, such as elastix [34] and Insight Segmentation and Registration Toolkit (ITK, Kitware Inc, New York, USA). In the future we plan to intigrate our proposed automatic method for quantifying PWV into MASS (LUMC, The Netherlands), to be used for research purposes.

## Conclusions

In conclusion, we have presented and evaluated a fully automatic approach that can compute aortic pulse wave velocity using cardiac MRI scans. We have also demonstrated that the proposed method has excellent agreement when compared to manual analysis. This indicates that the method has the potential to be used is clinical practice and would especially have a huge advantage when used in the context of large-population based studies.

## Abbreviations

*Δ**t*: transit time
MIP: Maximum intensity projection
MPR: Multi-planar reformatted
PWV: Pulse wave velocity
SENSE: Sensitivity encoding protocol
VE: Velocity encoded MRI
VENC: phase contrast velocity
